# Germline HOXB13 p.Gly84Glu mutation and cancer susceptibility: a pooled analysis of 25 epidemiological studies with 145,257 participates

**DOI:** 10.18632/oncotarget.5994

**Published:** 2015-10-17

**Authors:** Qiliang Cai, Xinpeng Wang, Xiaodong Li, Rui Gong, Xuemei Guo, Yang Tang, Kuo Yang, Yuanjie Niu, Yan Zhao

**Affiliations:** ^1^ Department of Urology, Tianjin Institute of Urology, the Second Hospital of Tianjin Medical University, Tianjin, 300211, China; ^2^ Department of Radiotherapy, the Second Hospital of Tianjin Medical University, Tianjin, 300211, China; ^3^ Pharmaceutical Department, the Second Hospital of Tianjin Medical University, Tianjin, 300211, China; ^4^ Library of Tianjin Medical University, Tianjin Medical University, Tianjin, 300070, China; ^5^ Tianjin Institute of infectious diseases, the Second Hospital of Tianjin Medical University, Tianjin, 300211, China

**Keywords:** HOXB13 gene, rs138213197, genetic mutation, cancer, risk

## Abstract

Numerous studies have investigated association between the germline HOXB13 p.Gly84Glu mutation and cancer risk. However, the results were inconsistent. Herein, we performed this meta-analysis to get a precise conclusion of the associations. A comprehensive literature search was conducted through Medline (mainly Pubmed), Embase, Cochrane Library databases. Crude odds ratios (ORs) and their 95% confidence intervals (CIs) were calculated by STATA 12.1 software to evaluate the association of HOXB13 p.Gly84Glu mutation and cancer susceptibility. Then, 25 studies including 51,390 cases and 93,867 controls were included, and there was significant association between HOXB13 p.Gly84Glu mutation and overall cancer risk (OR = 2.872, 95% CI = 2.121–3.888, *P* < 0.001), particularly in prostate cancer (OR = 3.248, 95% CI = 2.313–4.560, *P* < 0.001), while no association was found in breast (OR = 1.424, 95% CI = 0.776–2.613, *P* = 0.253) and colorectal cancers (OR = 2.070, 95% CI = 0.485–8.841, *P* = 0.326). When we stratified analysis by ethnicity, significant association was found in Caucasians (OR = 2.673, 95%CI = 1.920–3.720, *P* < 0.001). Further well-designed with large samples and other various cancers should be performed to validate our results.

## INTRODUCTION

Cancer is a serious problem endangering the human health and lives. Based on the reports from International Agency for Research on Cancer (IARC), cancer has become the second leading cause of mortality in developing countries, which has exceeded the mortality caused by cardiovascular incidences and become the leading cause of mortality in developed countries [1, 2]. Totally, 1,658,370 new cancer cases were diagnosed and 589,430 patients died from cancer in the United States in 2015 [3], suggesting that the burden of cancer will be heavier year by year, due to the increasing number of world population and the problem of aging is getting worse [4]. Although the mechanism of carcinogenesis remains elusive, multiple environmental and lifestyle factors has been confirmed contributed to the formation of cancers. However, not all cancer patients who have been exposed to the risk factors will develop cancer, suggesting the inter-individual differences in susceptibility [5]. Therefore, genetic, environmental and life time factors were suggested to be the main determinant of individual risk for cancer [6, 7]. In recent years, numerous studies have pointed out genetic factors, particularly single nucleotide polymorphisms (SNPs) of genes, plays crucial roles in tumorigenesis [8–11].

HOXB13, which encodes the transcription factor 13, belongs to the HOXB gene cluster at chromosome 17 [12], involves in embryonic development of different organs [13], regulates transcription of androgen receptor (AR) target genes [14] and is reported to function as a tumor suppressor in cancer [15]. Deregulation of HOXB13 expression has been reported in a number of malignancies, including prostate, breast, colon, lung, endometrial, renal cancers and melanoma [16–19]. Recently, a novel germline mutation, p.Gly84Glu (rs138213197), in exon one of the HOXB13gene, was suggested to have a close relationship with the risk of various cancers. Numerous studies have focused on the association between the germline HOXB13 p.Gly84Glu mutation and cancer risks, however, the results are inconsistent. Thus, we comprehensively searched all related literatures and performed present meta-analysis which has great power through polling all eligible related data to get a more precise conclusion.

## RESULTS

### Characteristics of included studies

**Figure 1:**
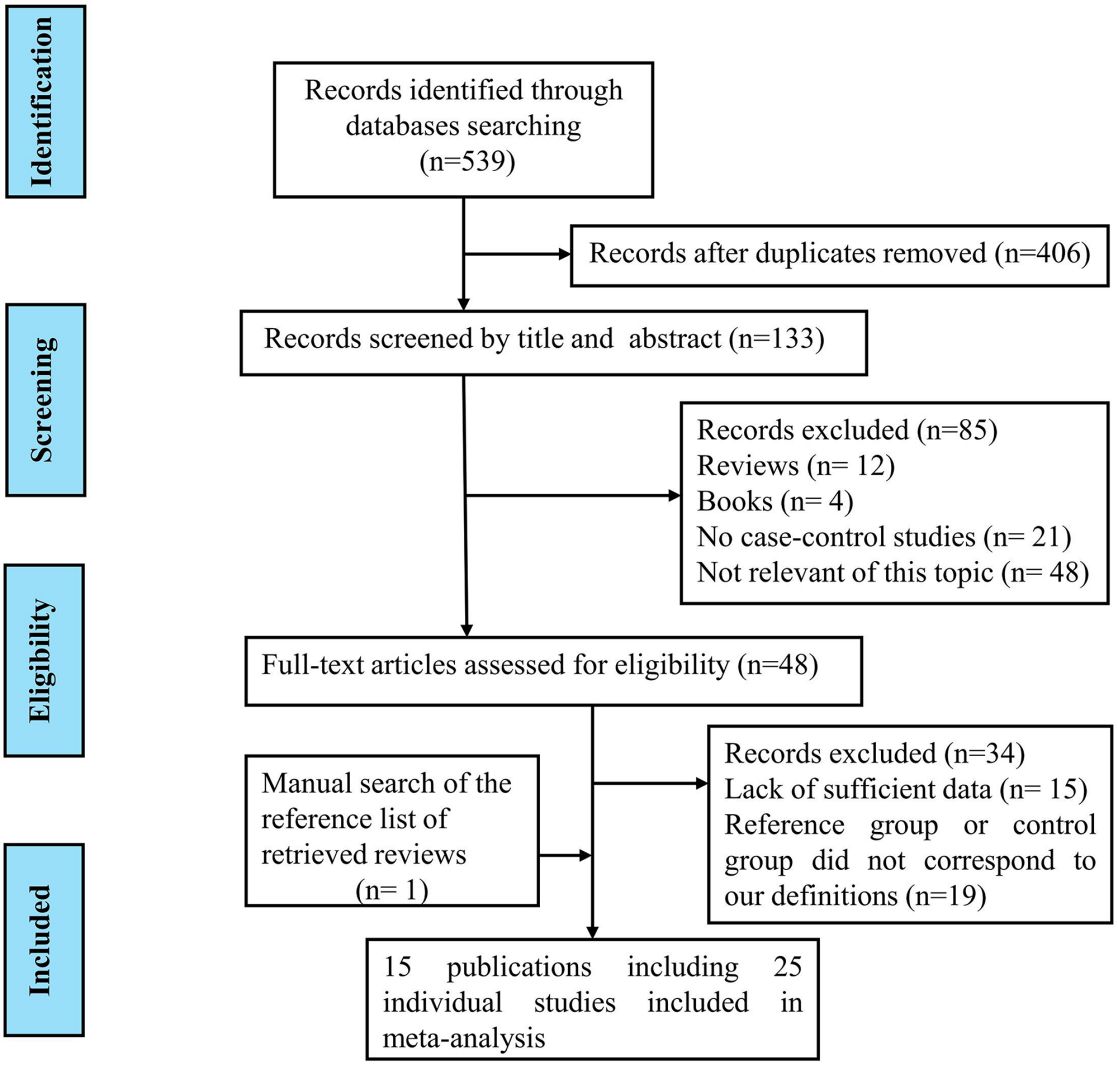
Flow diagram of summarizing the search strategy

Figure 1 represents the process of eligible studies' identification and selection. The literature selective process was conducted rigorously according to the inclusion and exclusion criteria. Finally, 15 publications involving 25 individual studies with 51,390 cases and 93,867 controls were included in the present meta-analysis [20–34]. The main characteristics of included studies were summarized in Table 1. These studies included 19 prostate cancer studies, 3 breast cancer studies and 3 colorectal cancer studies. OF the 25 studies, there were 21 studies of Caucasian, and the other four studies of mixed ethnicity (both of them were the mixed population of Caucasian, Asian and African-American). As for the control source, five studies applied population-based (PB) control, 14 studies employed hospital-based (HB) control, four studies applied PB/HB control, one studies applied family-based (FB) control, while the other one applied HB/FB control. Simultaneously, various genotyping methods were employed of all included studies, such as, 10 studies applied TaqMan assay, two studies applied Sanger sequencing, four studies applied MassARRAY iPLEX, six studies applied Illumina SNP, and the rest three studies employed complex methods (TaqMan, MassARRAY iPLEX, Sanger sequencing). The genotype distributions of all included studies in this meta-analysis were in agreement with Hardy-Weinberg equilibrium (HWE). The estimated quality of all included studies was in the range of 7–9 scores and was listed in Table 1.

**Table 1:** Characteristics of included studies in this meta-analysis

Study	Country	Ethnicity	Cancer type	Study design	Genotyping methods	Sample size		Cases		Controls		HWE	Score
						Cases	Controls	C	NC	C	NC		
Ewing 2012	USA	Caucasian	PC	HB	TaqMan	5083	2662	72	5011	4	2658	Yes	9
Breyer 2012	Mixed countries	Mixed	PC	HB	TaqMan	928	930	20	908	2	928	Yes	9
Akbari 2012	Mixed countries	Mixed	PC	HB	Sanger sequencing	1853	2225	10	1843	2	2223	Yes	8
Karlsson 2012^a^	Sweden (CAPS)	Caucasian	PC	PB	MassARRAY iPLEX	2805	1709	130	2675	24	1685	Yes	9
Karlsson 2012^b^	Stockholm-1	Caucasian	PC	HB	MassARRAY iPLEX	2098	2880	91	2007	37	2843	Yes	9
Gudmundsson 2012^a^	Chicago-SPORE	Caucasian	PC	HB	Illumina SNP chips	1988	1260	11	1971	5	1255	Yes	9
Gudmundsson 2012^b^	Iceland-ICR	Caucasian	PC	HB	Illumina SNP chips	4537	54444	13	4524	44	54400	Yes	9
Gudmundsson 2012^c^	The Netherlands	Caucasian	PC	PB/HB	Illumina SNP chips	1520	1916	23	1497	4	1912	Yes	9
Gudmundsson 2012^d^	Spain-Zaragoza	Caucasian	PC	HB	Illumina SNP chips	717	1692	1	716	0	1692	Yes	9
Gudmundsson 2012^e^	UK-ProtecT	Caucasian	PC	HB	Illumina SNP chips	561	1825	6	505	1	1824	Yes	9
Gudmundsson 2012^f^	Romania-Bucharest	Caucasian	PC	HB	Illumina SNP chips	722	857	1	721	1	856	Yes	9
Akbari MR 2012^a^	Canada	Caucasian	BC	HB	TaqMan	1804	925	2	1802	1	924	Yes	8
Akbari MR 2012^b^	Poland	Caucasian	BC	HB	TaqMan	2233	1837	5	2228	3	1834	Yes	8
Chen 2013	Mixed countries	Mixed	PC	HB	MassARRAY iPLEX	20	3887	7	13	701	3186	Yes	7
Xu 2013	Mixed countries	Caucasian	PC	FB	MassARRAY iPLEX	326	117	154	172	36	81	Yes	8
Kluzniak 2013	Poland	Caucasian	PC	PB	TaqMan	3515	2604	20	3495	3	2601	Yes	9
Laitinen 2013^a^	Finland	Caucasian	PC	PB/HB	Complex	4571	923	120	4451	28	895	Yes	9
Laitinen 2013^b^	Finland	Caucasian	BC	PB/HB	Complex	986	1449	16	970	16	1433	Yes	9
Laitinen 2013^c^	Finland	Caucasian	CC	PB/HB	Complex	442	459	7	435	0	459	Yes	9
Stott-Miller 2013	USA	Caucasian	PC	PB	TaqMan	1457	1442	18	1439	5	1437	Yes	9
Witte 2013	Mixed countries	Mixed	PC	HB/FB	TaqMan	1645	1019	20	1625	3	1016	Yes	8
Mohammad R. Akbari 2013^a^	Canada	Caucasian	CC	PB	TaqMan	1952	1197	11	1941	4	1197	Yes	8
Mohammad R. Akbari 2013^b^	Australia	Caucasian	CC	PB	TaqMan	743	246	2	741	1	245	Yes	9
Albitar F 2015	USA	Caucasian	PC	HB	Sanger sequencing	232	110	2	230	1	109	Yes	8
Kote-Jarai 2015	UK	Caucasian	PC	HB	TaqMan	8652	5252	134	8518	28	5224	Yes	8

a,b,c,d,e,f: represents different studies in one publication; NA: not available; PC: prostate cancer; BC: breast cancer; CC: colorectal cancer; HB: hospital based study; PB: population based study; FB: family based study; C: mutation carriers; NC: non mutation carriers; HWE: Hardy-Weinberg equilibrium.

### Quantitative data analyses

**Figure 2:**
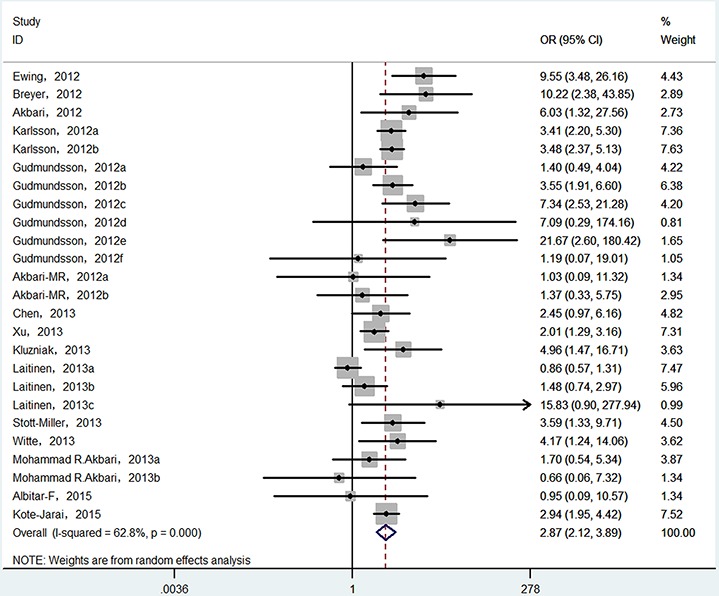
Forest plot of overall cancer risk associated with HOXB13 p.Gly84Glu mutation

**Figure 3:**
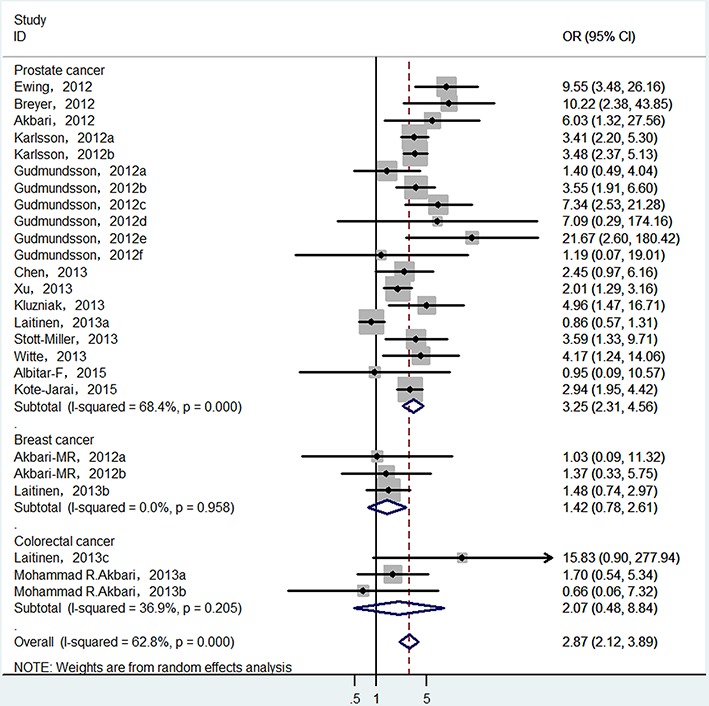
Forest plot of prostate cancer risk associated with HOXB13 p.Gly84Glu mutation

Finally, 25 epidemiological individual studies including 51,390 cases and 93,867 controls were enrolled in this meta-analysis. There is significant heterogeneity was found in over cancer risk estimation (*I^2^* = 62.8%, P_heterogeneity_ < 0.0001). Considering that, random-effects model was used to examine the association between HOXB13 p.Gly84Glu mutation and overall cancer susceptibility (OR = 2.872, 95% CI = 2.121−3.888, *P* < 0.001; Figure 2). In order to detect the source of heterogeneity, subgroup analyses were conducted by cancer type, ethnicity, control source and genotyping method. When we stratified by cancer type, there is significant heterogeneity was existed (*I^2^* = 68.4%, P_heterogeneity_ < 0.0001) and then random-effect models was used to verify the relationship between HOXB13 p.Gly84Glu mutation and prostate cancer risk. The results presented that HOXB13 p.Gly84Glu mutation contributed to the susceptibility of prostate cancer (OR = 3.248, 95% CI = 2.313−4.560, *P* < 0.001; Figure 3). While no heterogeneities (for breast cancer: *I^2^* = 0.0%, P_heterogeneity_ = 0.958; and for colorectal cancer: *I^2^* = 36.9%, P_heterogeneity_ = 0.205, respectively) were found when we analyzed the association between HOXB13 p.Gly84Glu mutation and the risk of the other two kinds of cancers mentioned above. HOXB13 p.Gly84Glu mutation was not contributed to the development of breast cancer (OR = 1.423, 95% CI = 0.774−2.615, *P* = 0.256; Table 2) and colorectal cancer (OR = 2.458, 95% CI = 0.98−6.177, *P* = 0.056; Table 2) using fixed-effect models. Moreover, further subgroup analyses were also performed by study design and genotyping method. All the results of meta-analyses were summarized in Table 2.

**Table 2:** Meta-analyses results of the association between germline HOXB13 p.Gly84Glu mutation and cancer risk

Variables	No.	Sample size	*P*_heterogeneity_	Analyzing model	OR	95% CI	*P* value
***Total***	25	145,257	<0.001	Random	2.872	2.121, 3.888	<0.001
***Cancer type***							
Prostate cancer	19	130,795	<0.001	Random	3.248	2.313, 4.560	<0.001
Breast cancer	3	9,423	0.958	Fixed	1.423	0.774, 2.615	0.256
Colotrectal cancer	3	5,039	0.205	Fixed	2.458	0.978, 6.177	0.056
***Ethnicity***							
Caucasians	21	144,007	<0.001	Random	2.673	1.920, 3.720	<0.001
Mixed decedents	4	12,507	0.362	Fixed	4.164	2.226, 7.790	<0.001
***Genotype method***							
TaqMan	10	46,126	0.149	Fixed	3.649	2.728, 4.880	<0.001
Sanger sequencing	2	4,420	0.201	Fixed	3.862	1.110, 13.441	0.034
MassARRAY iPLEX	4	13,842	0.201	Fixed	2.956	2.337, 3.740	<0.001
Illumina SNP chips	6	72,039	0.135	Fixed	3.934	2.479, 6.245	<0.001
Complex methods	3	8,830	0.067	Fixed	1.119	0.784, 1.597	0.537
***Source of control***							
Hospital based -HB	14	112,986	0.155	Fixed	3.363	2.449, 4.619	<0.001
Population based -PB	5	17,670	0.481	Fixed	3.196	2.234, 4.573	<0.001
PB/HB	4	11,494	0.001	Random	2.378	0.814, 6.952	0.113
Family based -FB	1	443	—	Fixed	2.015	1.286, 3.156	0.002
HB/FB	1	2,664	—	Fixed	4.168	1.235, 14.062	0.021

OR, Odds ratio; 95% CI, 95% confidence interval.

### Sensitivity analyses

**Figure 4:**
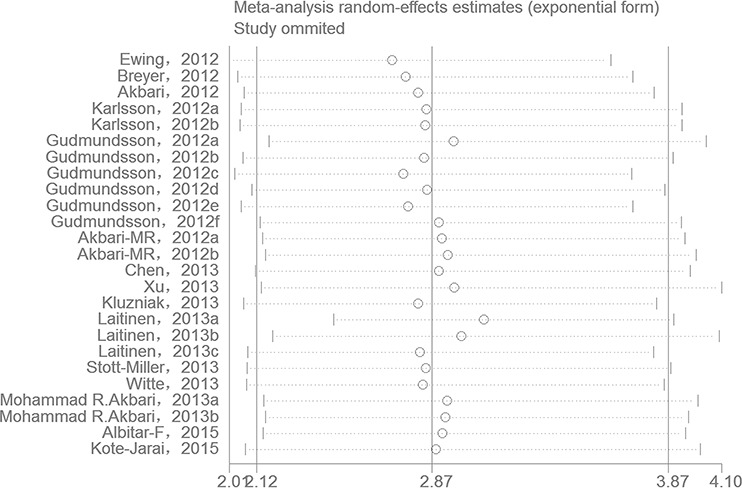
One-way sensitivity analysis of the pooled ORs and 95% CI for HOXB13 p.Gly84Glu mutation, omitting each data set in the meta-analysis

One-way sensitivity analysis of the pooled OR and 95% CIs for HOXB13 p.Gly84Glu was performed to verify if the results of our present meta-analysis were robust. The pooled ORs were calculated by means of a random effects model. To the best of our knowledge, in a sensitivity analysis, if a single study included in a meta-analysis was omitted each time, the pooled ORs were always persistent and it can be considered as the results of this meta-analysis were reliable and stable. In the present meta-analysis, no single study was qualitatively influenced by the pooled ORs when they were sequentially omitted, as indicated by the sensitivity analyses, suggesting that the results of our present study are stable (Figure 4).

### Publication bias

**Figure 5:**
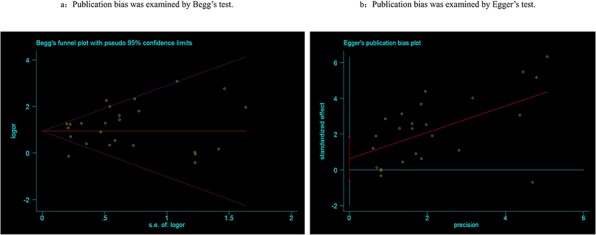
Publication bias was detected by Begg's (a) and Egger's (b) test

Begg's tests were employed to detect the potential publication bias that may be existed in this meta-analysis, and the results suggested there was no publication bias (*P* = 0.815, Figure 5a). Egger's tests also confirmed the absence of publication bias in the meta-analysis (*P* = 0.30, Figure 5b).

## DISCUSSION

Here, we conducted the largest meta-analysis to summarize the association between HOXB13 p.Gly84Glu mutation and cancer risk through pooling all candidate studies. Totally, 25 epidemiological case-control studies including 51,390 cases and 93,867 controls were enrolled this present study. In recent years, HOXB13 p.Gly84Glu mutation was found to contributed to the risk of prostate cancer [20–24, 26–31, 33, 34], especially in European countries [21]. However, no significant association was found in Asians [22] and Africans [21, 22]. Additionally, there are also three studies from two publications reported the association and they got the final conclusion that this mutation was not found to have increased the susceptibility of breast cancer on both familial and sporadic patients [25, 29]. Similarly, no significant association was detected among the mutation and the risk of colorectal cancer [29, 32]. In our present study, we summarized all the effects of HOXB13 p.Gly84Glu mutation with the risk of various cancers. We got a conclusion that the mutation is significantly increased the risk of cancers. Then, we performed subgroup analyses according to cancer type, ethnicity, text method, et al. We found that the mutation is contributed to the risk of prostate cancer, which was in accordance with previous studies. Moreover, we failed to find a statistical association between the mutation and the susceptibility of breast cancer and colorectal cancer. As such, the results were similar with the previous studies mentioned above. Moreover, stratified analyses by ethnicity were performed, and the conclusion suggested that the mutation can increase the risk of overall cancer among Caucasians, particularly in European decedent patients.

Substantial heterogeneity between studies was existed in our meta-analysis, just as a common aspect of genetic association studies. We determined the heterogeneity by Q-test and *I^2^* test, and statistically significant heterogeneity was observed. Then, random-effects model was used to analyze the ORs with 95% CI. Although we performed the present study according to the PRISAM strictly, including strict criteria of selection publications and meta-regression performance, there was no source of heterogeneity found in our present meta-analysis. Therefore, we carried out subgroup meta-analyses, and the results suggested that these parameters including ethnicity, cancer type, control source and genotyping method may be the main source of heterogeneities. In the stratified analysis by genotype method, the heterogeneity was significantly reduced, suggesting that genotype method may be one of source of heterogeneities.

To our knowledge, meta-analysis has great power through pooling all eligible studies, and thus gets a reliable and relative precise result. In the present study, there are several advantages existed. Above all, this is by far an analysis with the largest sample size, which can make our result more reliable and precise. What's more, the quality of each study include study was high, ranged from 7–9 score. Moreover, one-way sensitivity analysis was performed, and the result suggested that no significant influence of a single study on the pooled ORs and 95% CI. Simultaneously, no significant publication bias was detected in our present work. Of the two factors mentioned above, it demonstrated that our results were stability and reliable. In addition, subgroup analyses were conducted to explore the association of HOXB13 p.Gly84Glu mutation and susceptibility to the three types of cancers.

Some limitations existed and should be acknowledged in the meta-analysis. On the one hand, there were only three types of cancers including prostate cancer, breast cancer and colorectal cancers. Based on that, the results of this present work may not have enough power to represent all kinds of cancer. On the other hand, most of studies included in this meta-analysis were of European and USA decedents belonged to Caucasian ethnicity, which was a cause of selection bias, and other ethnicities, such as, African, and Asian ethnicities should be included in further studies. In addition, even though no sample size and language limitations were set, related studies in other languages may be ignored. What's more, only published studies were included in this meta-analysis, while other unpublished studies in different languages should be enrolled. Finally, adjusted estimations were not performed for insufficient data, such as, age, sex, smoking and drinking habits et al which can interrupt the results of present study. Simultaneously, interactions among gene–gene, gene-environment, and even different polymorphism loci of the same gene were not conducted for lacking of sufficient data in this work, which may regulate the gene expression, affect the function of gene product, and lead to the different OR values. Thus, further studies with same topics should consider the factors mentioned above.

In summary, the meta-analysis suggested that HOXB13 p.Gly84Glu mutation contributed to the overall cancer risk, especially for prostate cancer. Considering limitations mentioned above, further well-designed studies with larger sample size should be conducted to verify the results of the present meta-analysis.

## MATERIALS AND METHODS

The present meta-analysis was performed according to the latest meta-analysis guidelines (PRISMA) [35].

### Search strategy

A comprehensive computerized literature search was conducted through Medline (main Pubmed), Embase, Cochrane Library, Web of Science, Wanfang and China National Knowledge Infrastructure (CNKI) databases for related research studies reported the association between HOXB13 p.Gly84Glu mutation and cancer susceptibility. Furthermore, we also searched related studies manually from the references of reviews and articles reported the same topics. Combinations of searching terms were used as follows: “HOXB13 p.Gly84Glu”, “HOXB13 rs138213197”, “single nucleotide polymorphism, SNP or variation, mutation” and “cancer or carcinoma or tumor or neoplasms”. In order to get a precise conclusion, no sample size and language limitations were set, hoping that we can identify all the studies that examined the association of HOXB13 p.Gly84Glu mutation and cancer risk.

### Inclusion and exclusion criteria

The following criteria should be met of each included studies. (1) reported the association between HOXB13 p.Gly84Glu mutation and cancer risk; (2) used case-control design; (3) all the patients in cases group should be diagnosed by histochemical results or other gold diagnostic standers; (4) provided sufficient data of HOXB13 p.Gly84Glu mutation carriers or non-carriers, or other information such as ORs with 95% CIs for statistical analysis; (5) if there were several publications with overlapping data, only the latest one with the largest sample size was finally included in this present work. At the same time, if each of the searched studies was in accordance with the following criteria, it must be excluded. (1) human being studies; (2) review, meeting or other types of abstracts, comment, correspondence, letters or letters to the editor; case reports, or case-only studies; (3) not provided the sufficient data to extract.

### Data extraction

Two independent investigators extracted the essential information according to the selection criteria mentioned above. The key data was listed as follows: the first author's surname, year of publication, country of origin, ethnicity, cancer type, control source [population based (PB) or hospital based (HB)], the total number of cases and controls, genotyping methods, the number of HOXB13 p.Gly84Glu mutation carriers and non-carriers. Any disagreement was resolved by discussion, if not, other authors will join the discussion until consensus was reached.

### Statistical analysis

Crude odds ratio (OR) with corresponding 95% confidence interval (95% CI) were used to calculated to assess the strength of the association between the HOXB13 p.Gly84Glu mutation and cancer risk. Heterogeneity was evaluated by *I^2^* test, with the *I^2^* value ranged from 0 to 100%: *I^2^* = 0–25%: no heterogeneity; *I^2^* = 25–50%: moderate heterogeneity; *I^2^* = 50–75%: large heterogeneity; *I^2^* = 75–100%: extreme heterogeneity) [36, 37], and Cochrane Q test (*P* < 0.10 represents a significant heterogeneity was existed). Both fixed-effects (Mantel-Haenszel) and random-effect (Der Simonian and Laird) models were used to analyze the pooled ORs. The fixed-effects model was used when there was no heterogeneity; otherwise, the random-effects model will be used [38, 39]. Subgroup analyses were conducted based on cancer type, control source, and geographical region, to determine the source of heterogeneity. One-way sensitivity analyses were also performed to evaluate the influence of each included study to the results of present meta-analysis. An estimation of potential publication bias was carried out by the Begg's and Egger tests. All the key parameters were calculated using STATA software (version 12.0; Stata Corporation, College Station, TX, USA). All the tests were two-sided, a *P* value of less than 0.05 for any test or model was considered to be statistically significant.
